# My journey: from clinician to educator

**Published:** 2018-07-31

**Authors:** Wanjiku Mathenge

**Affiliations:** 1 Consultant Ophthalmologist and Director of Training and Research: Rwanda National Institute of Ophthalmology and Dr Agarwal's Eye Hospital, Kigali, Rwanda.

Just before my final year of residency training, the entire faculty from the University went on an industrial strike that would last a whole year. We were on our own as residents and yet I credit this year as the most productive in my training. Our seniors took on the role of educators and mentors, teaching me more surgical and administrative skills than I had learnt in my previous years. They were my role models. They made me realise that, as clinicians, we have a moral obligation to be educators. I have continued to cherish and invest in this role throughout my career.

In later years, as Head of Department of Ophthalmology in Rwanda, I realised that, as clinicians, we teach but have no training as educators. Being a great ophthalmologist does not make one a great ophthalmology educator!

I purposefully set about to improve my skills as an educator through online courses for educators, learning about curriculum development and how to evaluate different ophthalmology curricula for our local needs. At conferences I registered for education courses, and was lucky to benefit from a ‘Training the Trainers’ programme through a link between my College of Blending my clinical and education skills and knowledge has enabled me to design a curriculum that was benchmarked against international standards. I was able to design the Primary Eye Care curriculum for use by nurses in the WHO Africa region. In my quest to mentor a new generation, we have now launched the first ophthalmology residency programme in Rwanda using an innovative model that is untried in our region. I am glad that I have adequate skills to confidently navigate the world of competencies, mind maps, assessment tools such as Socrative or Kahoot, milestones and portfolios.

